# Case Report: Non-episodic Angioedema With Eosinophilia in a Young Lactating Woman

**DOI:** 10.3389/fimmu.2021.627360

**Published:** 2021-04-26

**Authors:** Mizuho Hirmatsu-Ito, Nobuhisa Nakamura, Megumi Miyabe, Tatsuaki Matsubara, Keiko Naruse

**Affiliations:** Department of Internal Medicine, School of Dentistry, Aichi Gakuin University, Nagoya, Japan

**Keywords:** edema, angioedema, eosinophil, cytokine, chemokine, case report

## Abstract

Angioedema with eosinophilia is classified into two types: episodic angioedema with eosinophilia (EAE), known as Gleich’s syndrome, and non-episodic angioedema with eosinophilia (NEAE). We present the case of a young lactating woman with non-episodic angioedema. She had no history of parasitic or nonparasitic infections. Physical examination showed striking, non-pitting edema in both lower extremities. Her weight had not changed significantly throughout the course of the illness. She exhibited no other symptoms, and her vital signs were normal. There was no evidence of anemia, hypoalbuminemia, thyroid dysfunction, heart failure, renal failure, or postpartum cardiomyopathy. Based on these findings, we diagnosed her with angioedema with eosinophilia. Given the scarcity of information about this condition, we explored the dynamics between cytokines/chemokines and edema in this patient. We successfully quantified the edema by bioimpedance analysis. In addition, we revealed the involvement of interleukin-5 (IL-5), thymus- and activation-regulated chemokine/C-C motif chemokine ligand-17 (TARC/CCL-17), eotaxin-3/CCL-26, tumor necrosis factor-α (TNF-α), vascular endothelial growth factor (VEGF), monocyte chemotactic protein-4/CCL-13 (MCP-4/CCL-13), eotaxin-1/CCL-11, and regulated on activation, normal T expressed and secreted/CCL-5 (RANTES/CCL-5) in NEAE. Lastly, we elucidated the strong association between these parameters. To the best of our knowledge, this is the first such study of its kind.

## Introduction

Angioedema is one of the causes of edema, first reported by Heinrich Quinche (1). Angioedema is classified into hereditary angioedema, allergic angioedema, drug-induced angioedema, idiopathic angioedema, physical angioedema, and angioedema with eosinophilia. Angioedema with eosinophilia is not difficult to diagnose due to the presence of marked eosinophilia, as long as it is considered as a differential. Angioedema with eosinophilia is classified into two types: episodic (EAE), known as Gleich’s syndrome, and non-episodic (NEAE) (2, 3). In most cases of EAE, patients present with fever, urticaria, and weight gain. In contrast, patients with NEAE are typically young females who are afebrile, report no weight gain, and have localized edema in the extremities (4). Compared with EAE, NEAE lacks serum IgM elevation and usually does not recur (3). NEAE is rather frequent in Japan than in Europe or America (5). The common feature between the two types is the lack of internal organ involvement (3). In some NEAE cases, therapeutic treatment with steroids, anti-IL-5 monoclonal antibodies, or anti-histamine has been effective (6, 7). Conversely, other studies showed that most patients with NEAE attained complete remission without corticosteroid therapy (4). However, there are few clear-cut guidelines to help distinguish the two, no valid treatment guidelines, and no indicators to assess the disease state.

Here, we would like to elucidate the dynamic interplay of cytokines/chemokines, and edema, and explain their involvement in the recovery of a young lactating woman suffering from acute severe edema, who recovered without pharmacological therapy. We also aim to explore the objective indications of this disease.

## Case Description

The patient was a 31-year-old lactating woman who had delivered her first child 4 months before. Her medical history included infantile asthma, and her family history revealed hyperuricemia and hypertension in her father, and Hashimoto’s disease in her mother.

Upon developing pruritis on bilateral lower limbs, she visited a dermatology clinic and was prescribed a steroid cream. One week after the onset of pruritis, she developed lower limb edema. She visited our hospital two weeks after the onset of edema. She reported no history of parasitic or non-parasitic infections.

**Figure 1:**
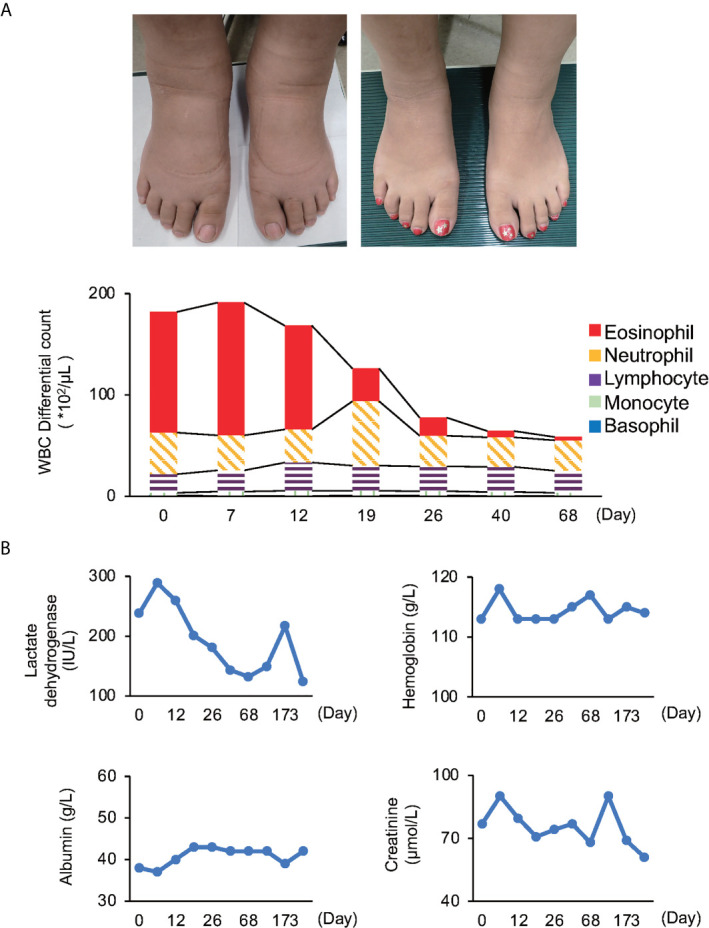
Time series of various examinations **(A)** White blood cell (WBC) differential count; Patient’s feet upon examination (right panel: edematous, left panel: recovery). Both white blood cell and eosinophil counts reached their peak on day 7. **(B)** Investigations: Lactate dehydrogenase was slightly elevated in the initial stage, but soon normalized. The reference range for LDH is 110-220 IU/L. Other investigations were within normal range through the course of illness.

Physical examination revealed striking, non-pitting edema of both lower legs and feet (**Figure 1A**). She did not have fever or urticaria. Her weight had not changed significantly. Otherwise, she showed no symptoms, and her vital signs were normal. Initial laboratory findings were as follows: white blood cell count was 1.82×10^4^/μL, and it increased to 1.91×10^4^/μL with 68.6% eosinophils later (eosinophil count was as high as 1.31×10^4^/μL) (**Figure 1A**). Serum lactate dehydrogenase was slightly elevated (**Figure 1B**). Serum immunoglobulin G (IgG), IgM, rheumatoid factor, antinuclear antibody, complement component 4, and erythrocyte sedimentation rate were within normal range. The maximum C-reactive protein level was only 0.51 mg/dL throughout the course of illness (**Supplementary Table 1**). There was no evidence of anemia, hypoalbuminemia, or thyroid dysfunction (**Figure 1B**).

Chest radiography showed no signs of heart failure or renal failure, and ultrasound cardiography was negative for postpartum cardiomyopathy.

Since she had edema with eosinophilia but no involvement of internal organs, we diagnosed her with angioedema with eosinophilia. She had no fever, urticaria, or weight gain. Serum IgM concentration was normal. Based on these findings, the final diagnosis was NEAE. She gradually recovered in approximately three months without any medication throughout the course. The follow-up period of 1 year was completely uneventful. Written informed consent was obtained from the patient.

**Figure 2:**
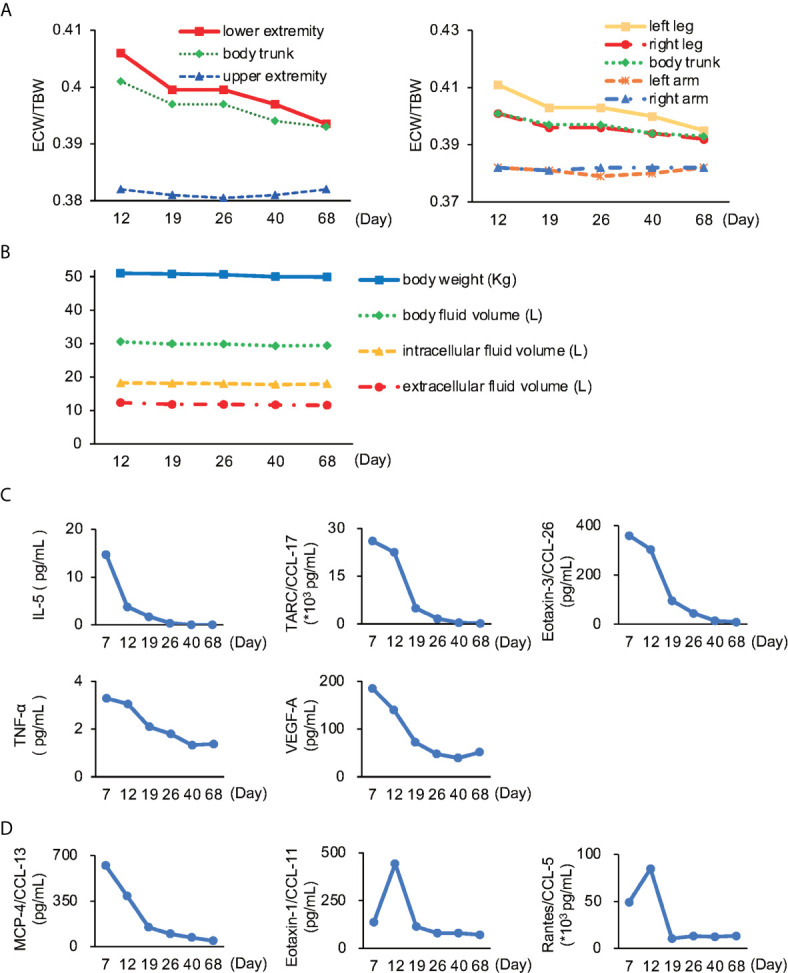
Body impedance analysis and cytokines/chemokines **(A)** The ratio of extracellular water to total body water (ECW/TBW). Lower extremity, especially left leg showed the most severe edema. The normal range for ECW/TBW for edema is less than 0.39 (8–11). **(B)** Time series of body weight and fluid volume in each part remained unchanged throughout the course of illness. **(C)** Cytokines and chemokines known to be involved in NEAE. IL-5, interleukin-5; TARC, thymus-and activation-regulated chemokine; CCL, C-C motif chemokine ligand; TNF-α, tumor necrosis factor-α; VEGF-A, vascular endothelial growth factor-A. **(D)** Cytokines and chemokines newly reported to be involved in NEAE. MCP-4, monocyte chemoattractant protein-4.

To measure the degree of edema objectively, we used bioimpedance analysis using InBody® (InBody, Seoul, Korea). The high ratio of extracellular water to total body water (ECW/TBW) in the trunk and lower limbs, especially on the left side, concurred with the finding of edema **(** **Figure 2A**). Although the total amount of body water and intra- and extra-cellular water remained unchanged (**Figure 2B**), bioimpedance analysis revealed edema of the trunk and lower legs.

Next, we investigated the role of various cytokines and chemokines. We found that interleukin-5 (IL-5), thymus- and activation-regulated chemokine/C-C motif chemokine ligand-17 (TARC/CCL-17), eotaxin-3/CCL-26, tumor necrosis factor-α (TNF-α), and vascular endothelial growth factor (VEGF) showed correlation with the extent of disease (**Figure 2C**). Furthermore, we found the relationship between monocyte chemotactic protein-4/CCL-13 (MCP-4/CCL-13), eotaxin-1/CCL-11, and regulated on activation, normal T expressed and secreted/CCL-5 (RANTES/CCL-5), and the extent of disease (**Figure 2D**).

We explored the correlation between the index of edema (ECW/TBW) and these cytokines/chemokines. We found that each parameter we measured had a positive correlation with ECW/TBW. In particular, eotaxin-3/CCL-26, eotaxin-1/CCL-11, TARC/CCL-17, and MCP-4/CCL-13 were highly correlated (ρ value>0.9) with the index of edema (**Table 1**). In contrast, VEGF and RANTES/CCL-5 demonstrated a weak positive correlation (ρ value <0.5) with ECW/TBW.

**Table 1:** Correlation between cytokines/chemokines and ECW/TBW.

Cytokine/chemokine	ρ	p
VGEF-A	0.400	0.374
TNF-α	0.782	0.046
IL-5	0.887	0.008
TARC/CCL-17	0.927	0.003
Eotaxin-3/CCL-26	0.927	0.003
RANTES/CCL-5	0.426	0.328
MCP-4/CCL-13	0.927	0.003
Eotaxin-1/CCL-11	0.982	<0.001

## Discussion

In this study, we successfully demonstrated a correlation between edema and cytokines/chemokines in NEAE. Since no valid treatment guidelines are currently available for NEAE, we explored objective indices to evaluate the severity of edema and the disease.

Quantification of edema severity is challenging. However, in our patient, body impedance analysis revealed the exact site and extent of edema. We found that ECW/TBW could objectively evaluate the severity and site of edema. Specifically, the ECW/TBW values reflected the disease condition.

A previous study suggests that the possible pathogenesis of angioedema with eosinophilia occurred *via* IL-5 derived from activated T helper type-2 (Th-2) cells (12). Infiltrated eosinophils in dermal tissue induce LtCC4 and MBP secretion leading to histamine release from activated mast cells and basophils (2, 3). We found that IL-5 correlated with the extent of disease in our case. Motegi *et al.* observed that the number of eosinophils was reflected not only by TARC/CCL-17, but also by eotaxin-3/CCL-26, and VEGF. These results are consistent with our findings (13). Another study of three NEAE patients suggested that TNF-α is elevated in NEAE, but not in EAE (14). However, in our case, the level of TNF-α also indicated the disease state, and the TNF-α correlation with edema was higher than that of VEGF.

We further examined the relationship between various cytokines related to Th-2 cells and eosinophils by investigating the involvement of RANTES/CCL-5, eotaxin-1/CCL-11, and MCP-4/CCL-13 during the course of NEAE. Th-2 cells predominantly express CC-chemokine receptor (CCR) 3, CCR-4, and CCR-8 (15, 16). CCR-3, which is not only expressed by Th-2 cells, but also by eosinophils and basophils, has an affinity for eotaxin-3/CCL-26, MCP-4/CCL-13, eotaxin-1/CCL-11, and RANTES/CCL-5 (17, 18). CCR-3 is strongly expressed on peripheral eosinophils, and its agonist has a robust effect on eosinophil migration. CCR-4 has an affinity for TARC/CCL-17 (19). We found that the dynamics of MCP-4/CCL-13 were consistent with the severity of edema and eosinophilia. To the best of our knowledge, this is an unprecedented finding.

In our study, we demonstrated that multiple indices are associated with edema and eosinophilia. We found that most of the measured cytokines/chemokines had a positive relationship with ECW/TBW, with a ρ value of greater than 0.7.

Eotaxin-1/CCL-11 is an agonist for CCR3 and an antagonist for CCR2 (20). In contrast, MCP-4/CCL13 is an agonist for both CCR3 and CCR2 (21, 22). Moreover, CCR3 agonists, such as eotaxin-1/CCL-11, MCP-4/CCL13, or eotaxin-3/CCL26, strongly induce migration and degranulation in eosinophils (23). In basophils, the CCR3 agonists strongly induce migration in the same way that they do in eosinophils. CCR2 agonists such as MCP-4/CCL13 strongly induce degranulation, resulting in histamine release (22, 24, 25). The chemokines newly reported in our study, in particular, eotaxin-1/CCL-11 and MCP-4/CCL-13 were strongly associated with ECW/TBW in NEAE, suggesting they may prove to be useful for assessing NEAE severity. Eotaxin-1/CCL-11 and RANTES/CCL-5 reached their peak levels after eosinophils did.

In our study, RANTES/CCL-5, which is induced by TNF-α, showed a low correlation with edema. TNF-α–induced RANTES/CCL-5 production is not affected by Th-2 cells but is markedly enhanced by Th-1 cells (26). Eotaxin-1/CCL-11 is also upregulated by TNF-α (27). Consequently, regulation by TNF-α may be a reason for the delayed peaks of these two chemokines.

In addition to edema, which is the primary clinical symptom of NEAE, the primary histological feature of the disease is a granulomatous reaction in skin lesions (13, 28). Granulomas predominantly consist of macrophages and monocytes. Monocytes express CCR2, but not CCR3. Among the chemokines positively related to ECW/TBW, MCP-4/CCL13 is the only chemokine whose receptor is CCR2.

Previous studies have reported the remission of NEAE within 2-3 months of treatment with corticosteroids or antihistamines (29–31). In our patient, the peak eosinophil count was over 13000/μL. Notably, previously reported NEAE studies have demonstrated a wide variance in the number of eosinophils. Some patients with eosinophil counts less than 10000/μL received medical interventions (4, 6, 7), whereas some patients with eosinophil counts more than 10000/μL did not (4) (**Table 2**). In this case, remission was achieved within 3 months of onset without any pharmacological intervention. Cytokine/chemokine levels normalized within 1 month, with most showing a significant decrease in the first 19 days. Because the cytokine/chemokine levels reached a normal range before the eosinophils did, they could be considered possible indices of NEAE recovery. Unfortunately, we were unable to identify the very indications to determine treatment strategy, and therefore further studies will be required in this field. Based on our experience with this rare condition, treatment of NEAE may not always require drug intervention, as long as careful monitoring of internal organs and specific indices. Our study suggests that ECW/TBW, eotaxin-1/CCL-11, and MCP-4/CCL-13 could become key parameters for guiding treatment decisions.

**Table 2:** The principle findings in previous studies on NEAE.

References	Cases	Clinical or histological features	Treatments
**Chikama** **et al.** (3)	37 women with a mean age of 26 (20-37) years	The peak eosinophil counts were 6,480-11,203/μL. NEAE patients have following 4 common features: 1; the absence of recurrent attacks, 2; the predominance of young females, 3; the localization of the angioedema to the extremities, 4; the absence of increase on the serum IgM level.	8 cases took steroid therapy,13 cases took anti-histamine agent, and 2 cases received both medications. Spontaneous remission was observed in 13 cases. One case was not mentioned as to therapy in detail.
**Okamoto** **et al.** (29)	4 women with a median age of 25.5 years	Plasma levels of TARC/CCL17 in NEAE patients decreased according to the reduction in symptoms and eosinophilia.	One patient received no medical intervention. There is no mention of the other three.
**Nakachi** **et al.** (4)	11 women with an age of 28.5 ± 5.3 (23-38) years	The peak eosinophil counts were 7,839 ± 6,008 (2,130-23,170)/μL without an increase in the count of other leukocyte series. Edema was observed in distal extremities symmetrically. No internal organ involvement or no recurrence was observed in all the patients. Serum CRP and IgE levels stayed almost normal. The duration of edema was 5.2 ± 2.8 (range: 2-12) weeks.	3 patients received steroid therapy. The other 8 patients did not require steroid therapy.
**Takizawa** **et al.** (31)	13 women and 2 men, with a median age of 27 (25-31.5) years	The peak eosinophil counts were 8,910 (7,581-13,374)/μL. Observed edemas were in both lower limbs. No internal organ involvement was recorded. All patients achieved complete remission 6-9 weeks after onset without recurrence.	Antihistamine agents were administered to 10 patients, but no significant difference was observed between those with and without medication. None of the 15 patients required corticosteroids.
**Teraki** **et al.** (30)	3 women with a range of 21-31years	The peak eosinophil counts were 4,922-7,336/μL. Serum TARC levels were markedly elevated and correlated with peripheral blood eosinophil counts during PSL treatment.	Treatment with PSL improved edema in three patients.
**Goto** **et al.** (28)	12 women with a mean age of 27.8 (24-42) years	The peak eosinophil counts were 5,525 ± 3,017 (1,174-10,730)/μL. They have 3 patterns of histological feature: 1; eosinophilic granulomatous panniculitis (7 cases), 2; eosinophilic dermatitis without granulomatous reaction (3 cases), 3; invisible dermatosis (2 cases) The symptom duration of all patients was 5-8 weeks.	7 patients underwent a short term of oral corticosteroid therapy.
**Chu** **et al.** (6)	41-year-old woman	The peak eosinophil count was 1300/μL. The symptom duration was about 7 months.	Administration of intravenous and oral corticosteroid followed by anti-IL-5 monoclonal antibody administration.

## Data Availability Statement

The original contributions presented in the study are included in the article/**Supplementary Material**. Further inquiries can be directed to the corresponding author.

## Ethics Statement

Ethical review and approval was not required for the study on human participants in accordance with the local legislation and institutional requirements. The patients/participants provided their written informed consent to participate in this study. Written informed consent was obtained from the individual(s) for the publication of any potentially identifiable images or data included in this article.

## Author Contributions

MH-I conceptualized, investigated, administered, and visualized this project, NN curated the data, MM managed the resources, and MH-I drafted the manuscript, which was reviewed and edited by KN and approved by all coauthors. TM supervised all the authors. All authors contributed to this article and approved the submitted version.

## Conflict of Interest

The authors declare that the research was conducted in the absence of any commercial or financial relationships that could be construed as a potential conflict of interest.
