# Supplementary material for: Case Report: Non-episodic Angioedema With Eosinophilia in a Young Lactating Woman
Source: Front Immunol. 2021 Apr 26;12:627360. doi: 10.3389/fimmu.2021.627360 (PMC8107285; doi:10.3389/fimmu.2021.627360)
Supplement: Supplementary file 1 [file Presentation_1.pptx]

## Slide 1
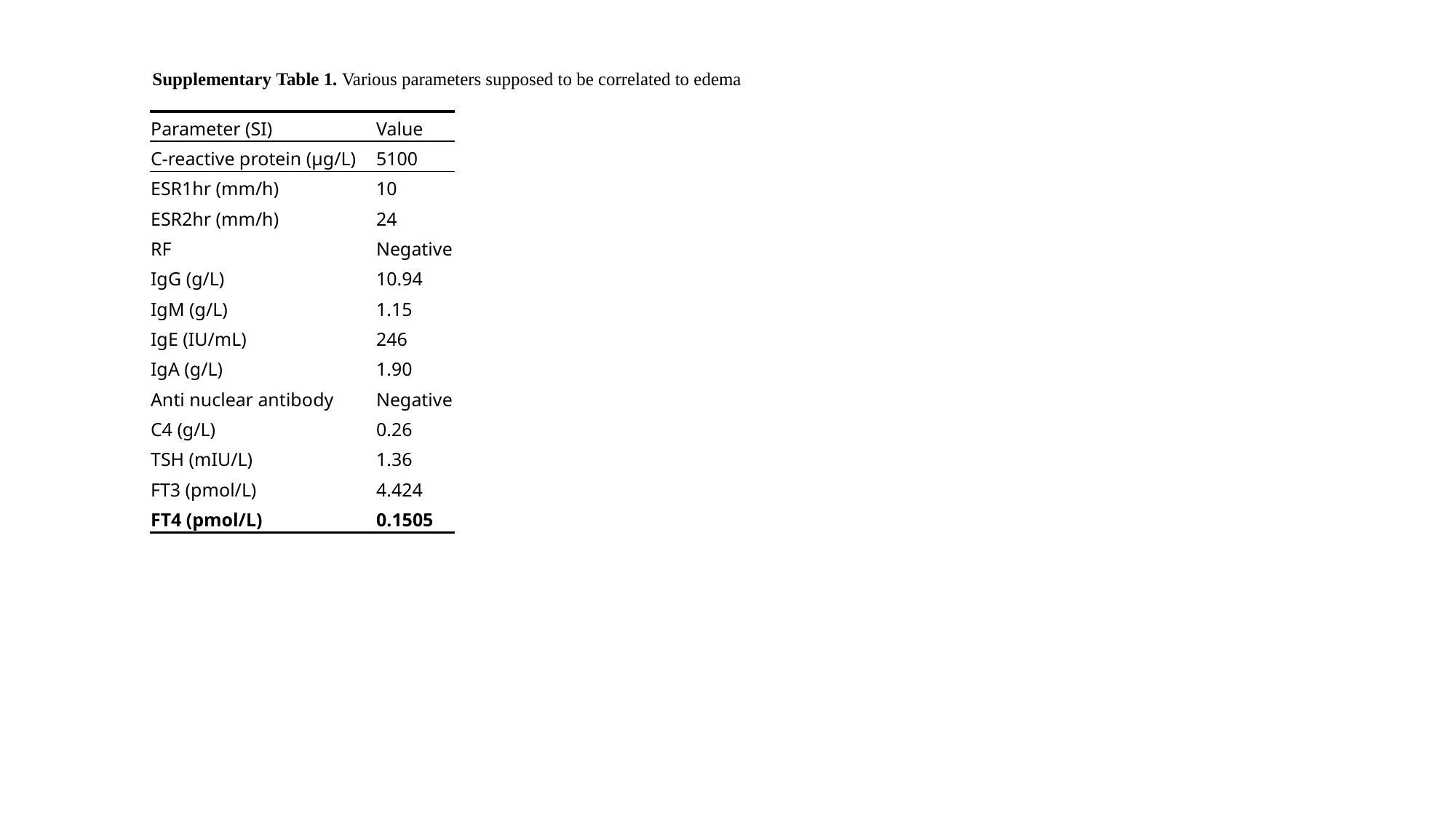

Supplementary Table 1. Various parameters supposed to be correlated to edema
| Parameter (SI) | Value |
| --- | --- |
| C-reactive protein (μg/L) | 5100 |
| ESR1hr (mm/h) | 10 |
| ESR2hr (mm/h) | 24 |
| RF | Negative |
| IgG (g/L) | 10.94 |
| IgM (g/L) | 1.15 |
| IgE (IU/mL) | 246 |
| IgA (g/L) | 1.90 |
| Anti nuclear antibody | Negative |
| C4 (g/L) | 0.26 |
| TSH (mIU/L) | 1.36 |
| FT3 (pmol/L) | 4.424 |
| FT4 (pmol/L) | 0.1505 |
